# Feasibility of a Non-Anticipatory, Random-Action Target System to Improve Shooting Performance: A Brief Field Trial

**DOI:** 10.3390/sports12110305

**Published:** 2024-11-11

**Authors:** Matthew Lee Smith, Ali Boolani

**Affiliations:** 1 School of Public Health, Texas A&M University, College Station, TX 77808, USA; 2 Human Performance and Nutrition Research Institute, Oklahoma State University, Stillwater, OK 74078, USA; ali.boolani@okstate.edu; 3 Department of Physiology and Pharmacology, Center for Health Sciences, Oklahoma State University, Tulsa, OK 74107, USA

**Keywords:** firearm training, shooting performance, shooting accuracy, random-action target system

## Abstract

Firearm shooting performance training rightfully focuses on shooting accuracy; however, additional foci should include decision processing speed and reaction time associated with decision making to avoid reaction-only based shooting responses. While advancements in realistic training environments attempt to mimic “real-world” situations, many remain largely anticipatory or subject to a speed–accuracy trade-off (SAT). The purpose of this brief field trial was to demonstrate the feasibility of a random-action target system (RATS) on participants’ shooting performance (i.e., accuracy, omission, and commission rates) among a convenience sample of six retired police officers and competitive shooters (age range: 45–58 years, mean age = 52.5 ± 5.89). Observational data were gathered from a single-day, three-round trial to test shooting accuracy and shooting errors when shooters were unable to anticipate target appearance location and target exposure speed. In Trial 1, the target exposure time was 0.5 s, which increased to 0.7 s in Trial 2, and decreased back to 0.5 s in Trial 3. Shooting accuracy generally increased, while omission and commission generally decreased, when shooters were presented with targets exposed for longer durations. From Trial 1 to Trial 3 (both trials with 0.5 s target exposures), shooters showed higher median accuracy rates, lower median omission rates, and lower median commission rates. Findings suggest that a non-anticipatory, RATS holds promise for improving shooting performance and offset SAT among shooters with firearm experience. However, additional trials are needed with the RATS to replicate these findings among a larger and more diverse set of participants, who train with the RATS consistently, over longer durations.

## 1. Introduction

Firearm shooting performance is important for law enforcement, competitive shooters, and military personnel. While shooting accuracy is paramount among competitive shooters [1], psychological factors such as confidence, concentration, and anxiety control can negatively impact shooting performance [2,3,4,5] and result in ‘choking’ episodes [6]. These psychological factors are also present in the real-world situations in which law enforcement and military personnel must discharge their firearm, requiring potentially “life-and-death” decisions made within an instant. Therefore, in addition to a focus on accuracy, law enforcement and military personnel must also consider the consequences of their actions or inactions in terms of accidental shooting errors (e.g., involuntary fire, friendly fire) [7,8,9,10,11,12,13,14] and shooting hesitancy (i.e., not firing when required) [5,15], respectively. Accidental shooting errors have been ascribed to the possibility of perceptual failures (e.g., confusing ‘friendly’/non-lethal targets for enemy/lethal targets) and may be a result of a response-strategy perspective [16,17,18,19,20]. As such, law enforcement and military personnel are faced with a speed–accuracy trade-off (SAT) when making decisions to fire their weapons [15]. The processing speed or reaction time associated with making these decisions is critical because shooting response delays may result in personal harm or harm to their partners, whereas reaction-only based shooting responses may result in hitting unintended targets or shooting inaccurately. Because shooting inaccuracy and ‘choking’ increase when shooters are in high-pressure situations that deviate from “performing routine processes” [21], new and innovative tactical firearms training approaches may be needed to mitigate the SAT and strengthen shooters’ abilities to process stimuli quickly and react with accuracy.

Most tactical firearms training involves stationary non-threatening targets that do not vary from test to test [22]. Such trainings commonly emphasize firearm safety, tactical knowledge, and marksmanship [22,23], which may be inadequate for mastery [22,24] and do not translate into “real-world” scenarios or confrontations. Even when attempts are made to enhance the ecological validity of training environments [22], it remains challenging to re-create the random nature of threating and non-threatening targets (e.g., when they appear, how long they appear for, their movements, their sizes) that mimic “real-world” situations experienced in the field. Recent efforts have advanced realistic training to incorporate dynamic, stress-inducing, and sometimes live-acted, scenarios and technology-based simulations [25,26,27,28,29,30,31,32] that may emphasize tactical decision making [17,33], risk management [34], and reaction times [35,36]. However, such trainings are brief and costly (i.e., time and human capital intensive), and limit consistent training exposure, which may render them less effective to offset the SAT in high-stress situations in the field.

In many training scenarios, humans develop a prepotent motor response as they struggle to fulfill the task requirements of responding as fast and accurately as possible in environments with constant cognitive loads [37]. That is, individuals training in environments under constant cognitive loads may create a semi-autonomous motor program, which may be executed quickly with minimal conscious interference, making the response the default action state (e.g., shooting at a target). Once individuals become aware of a stimulus, they can either choose to respond quickly (decision speed), or they can choose to fully process the stimuli prior to responding (decision accuracy). To achieve decision accuracy, individuals must display response inhibition [20], which may be challenging in a cognitively demanding environment [18,20]. Many training methods exhibit difficulty simulating environments capable of being cognitively demanding while requiring participants to display response inhibition [22]. Thus, to implement tactical firearm training that prepares shooters for the complexity of “real-world” scenarios, it is imperative that training environments are cognitively demanding, require response inhibition, and demand the shooter to maintain target accuracy.

**Figure 1:**
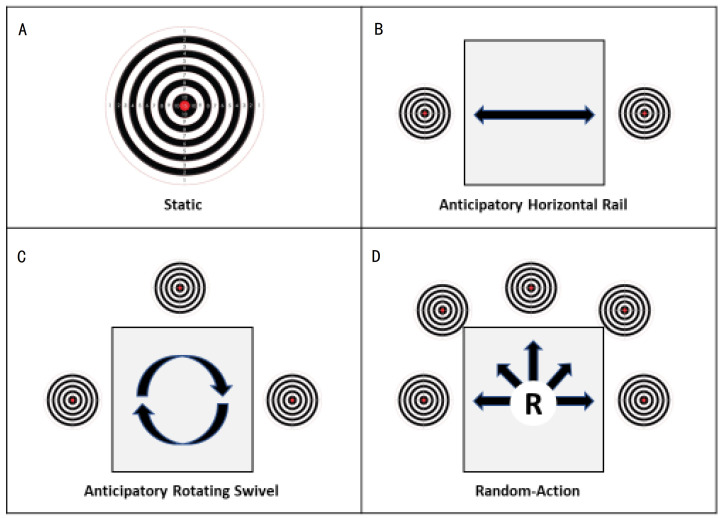
(**A**–**D**) Graphical examples of anticipatory and random-action targets.

The cognitive task of balancing between decision speed and decision accuracy, which can exacerbate the SAT, is further intensified in “real-world” situations by random target movement and other unknown variables (e.g., target size, target distance, target exposure duration). While training systems and simulations exist that move at set or randomized times, they typically do not encompass the other necessary training aspects that include simple attention reaction tasks, or other ecological variabilities, to train shooters for “real-world” applications. Therefore, there exists a need for a novel shooting system that may facilitate rigorous training scenarios that can better prepare individuals entering competition or possible combat situations. Examples of common static and anticipatory targets are provided in Figure 1. Additionally, Figure 1 illustrates a random-action target system (RATS), which randomly presents targets at various positions visible to shooters, from behind cover at various speeds. The RATS represents a departure from traditional training methodologies by prioritizing task inhibition and is the focus of this brief feasibility field trial. This system utilizes randomized target presentations, varying in size, distance, and speed, to challenge shooters to react swiftly and accurately to changing stimuli. By consistently exposing personnel to unpredictable scenarios, the RATS is believed to promote the development of rapid decision-making skills and enhance overall shooting proficiency.

In this context, the purpose of this brief feasibility field trial was to demonstrate the feasibility of a RATS on participants’ shooting performance (i.e., accuracy, omission, and commission rates) among a convenience sample of retired police officers and competitive shooters. Competitive shooters and retired police officers were chosen because of their years of firearms training, thus allowing the research team to observe the reaction to, and impact of, this randomizing, non-anticipatory shooting intervention.

## 2. Materials and Methods

### 2.1. Random-Appearance Target System (RATS)

As previously stated, the RATS is a system that randomly presents targets at various positions visible to shooters, from behind cover at various speeds. The system is battery powered and software controlled so the user can preset customizable settings that control the speed and/or the placement of the target. The RATS can be programmed for random target placement based on an X, Y axis operation and variable speed motor. There are six points of variability, which include: (1) target sizes and their relation to cover; (2) how and when the system is activated; (3) the motion profile (i.e., preset or completely random); (4) the speed of target movement; (5) the time targets remain covered before exposure; and (6) the time between target exposure and retraction to cover. The RATS was developed as a novel system to improve reaction time, increase shooting accuracy, and promote adaptability to dynamic environments. By systematically training shooters to react quickly and decisively to unpredictable stimuli, the RATS can facilitate the development of enhanced reaction time capabilities. The emphasis on real-time response may foster greater precision and target acquisition, resulting in higher hit rates and improved operational outcomes. Further, shooters trained with the RATS may be better equipped to navigate complex and rapidly evolving situations, ensuring readiness in diverse operational contexts.

### 2.2. Subjects

A convenience sample of nine firearm owners were recruited to participate in this brief feasibility field trial. The inclusion criterion for the trial was potential participants who had some level of firearm or tactical training, which excluded armature shooters. Participants were identified through the researchers’ social networks and were intended to vary in terms of their firearm- and tactical-related training and skill levels. A total of six participants were recruited, who were either retired police department (PD) (n = 3) or competition shooters (CS) (n = 3). Participants were classified as beginner (shooting at a basic level of proficiency without any certifications), intermediate (certification of proficiency), or advanced (certification of proficiency and advanced training). Participants were invited to attend a single-day, two-hour field trial of a novel RATS. After agreeing to participate, participants convened at an outdoor gun range. Participants were asked to bring their own handguns, ammunition, and personal protection equipment (i.e., protective eyewear and hearing protection). Participants were welcome to bring multiple handguns to the field trial, if desired. Once on-site, participants were introduced to the RATS and received specific instructions about the three-round trial protocol. General firearm safety instructions were provided, and participants were encouraged to ask clarifying questions. Participation in this field trial was voluntary, and participants could choose to discontinue at any time. No incentives were provided to participants. A review of this external evaluation protocol was performed by the Texas A&M Institutional Review Board (#2024-1180).

### 2.3. Trial Procedures

The purpose of the field trial was to test shooting accuracy and shooting errors when shooters of varying training and skill levels were unable to anticipate target appearance location. The three-round trial protocol required each participant to fire all rounds in a single magazine at random side-to-side and vertical target exposures by the RATS (see image D in Figure 1). To gage variation in reaction time, the trials differed based on the duration each target was exposed. In Trial 1, the full target exposure time for each randomly appearing target was 0.50 s. In Trial 2, the full target exposure time for each randomly appearing target was 0.70 s (i.e., 0.2 s longer duration for each target exposed). Trial 3 mirrored Trial 1, where the full target exposure time for each randomly appearing target was 0.50 s. The differing target exposure times between trials was intended to allow researchers to identify variation in shooting accuracy and reaction time during a shooting exercise that omits any element of target location anticipation. For all three trials, the time between target exposures was random, with non-predicable increments between one and three seconds (e.g., a sequence of 1.0, 1.2, 2.7, 3.0, 3.0, 1.4, 1.0 s between target exposures).

The RATS was positioned nine yards from the firing line. Before each trial, participants were instructed to load their firearms of choice, place their weapon on the table, and await further instruction. One at a time, participants were asked to pick-up their firearm and step to the firing line. Participants were instructed to fire at the RATS each time a target was randomly exposed, to the best of their ability, and continue firing at the RATS until their magazine was empty. Once the firearm magazine was empty, participants were asked to remove the magazine, place their weapon on the table, and await further instruction.

### 2.4. Data Collection

Data were collected by a team member who observed each participant during each trial. For each participant during each trial, this team member documented: (1) the number of times a target was exposed; (2) the number of times the participant discharged their firearm; (3) the number of times the participant hit the exposed target; and (4) the number of times a target was exposed but the participant did not discharge their firearm. These data were recorded by hand using paper–pencil during the trial, then entered into an Excel spreadsheet at a later time. Using these data points, shooting accuracy (i.e., percent of shots fired that hit the intended target), shooting omission (i.e., percent of shots not taken based on the number of opportunities to hit a target), and shooting commission (i.e., percent of times a target was not hit regardless of inaccuracy or omission) rates were calculated.

### 2.5. Data Analyses

All data transformations and analyses were performed using SPSS version 29. Shooting performance was measured as accuracy, omission, and commission rates, which were calculated for each participant for each trial round. To calculate shooting accuracy, we divided the number of times the participant hit the exposed target by the total number of times the participants discharged their firearm (i.e., [hits/attempts]). Accuracy rates ranged from 0% to 100%, with higher percentages indicating higher shooting accuracy. To calculate shooting omission, we divided the number of times the participants discharged their firearm by the total number of times a target was exposed, then subtracted that number from 1 (i.e., 1—[attempts/possible]). Omission rates ranged from 0% to 100%, with higher percentages indicating more errors of omission. To calculate shooting commission, we divided the number of times the participants hit the target by the total number of times a target was exposed, then subtracted that number from 1 (i.e., 1—[hits/possible]). Commission rates ranged from 0% to 100%, with higher percentages indicating more errors of commission.

Individual-level data for all six participants are presented by trial in terms of accuracy, omission, and commission. Medians and interquartile rankings (IQR; 25% and 75%) are presented for the group of participants by trail and shooting metric. Due to the exploratory nature of this study, an a priori power analysis could not be completed to determine sample size. Further, given the small sample size in this feasibility trial, tests of normality were not performed because the sample may not have captured the full range of variability in the data, potentially increasing the risks for Type I and Type II errors in the tests of normality. Consequently, non-parametric tests (i.e., Wilcoxon sign-rank tests) were utilized in this study. Changes in the proportion of participants who changed in terms of accuracy, omission, and commission were assessed between trials using Wilcoxon sign-rank tests. *p*-values <0.05 were used to identify statistically significant differences.

A post hoc effect size was calculated using the following formula η^2^ = Z^2^/(N) [38] using G*Power [39]. An effect size of 0.680 was calculated for the scores that were significantly different, using alpha = 0.05, 1 − B = 0.80. A sample size of 22 would be needed to complete a more robust single-arm trial.

## 3. Results

**Figure 2:**
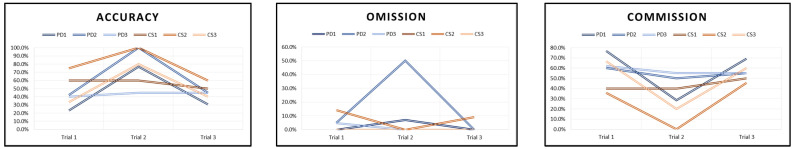
Accuracy, omission, and commission by participant.

Table 1 presents participant characteristics and shooting performance across trials. Shooting performance is also depicted graphically in Figure 2. Six male (age range = 45–58 years) participants completed the study, of which three were PD and three were CS. Five of the participants were classified as advanced shooters (n = 3 PD, n = 2 CS), with one participant being classified as an intermediate shooter. In terms of accuracy, the median accuracy rate was 41.1% in Trial 1, which increased to 78.5% in Trial 2 when target exposure times increased. The median accuracy rate subsequently decreased to 45.0% in Trial 3 when target exposure times returned shorter durations (i.e., same target exposure times as Trial 1). In terms of omission, the median omission rate was 2.4% in Trial 1, which decreased to 0.0% in Trial 2 when target exposure times increased. The median omission rate remained at 0.0% in Trial 3 when target exposure times returned shorter durations. In terms of commission, the median commission rate was 61.0% in Trial 1, which decreased to 34.3% in Trial 2 when target exposure times increased. The median commission rate increased to 55.0% in Trial 3 when target exposure times returned shorter durations.

Despite the consistent target exposure times in Trials 1 and 3, median accuracy rates increased (41.1% vs. 45.0%), median omission rates decreased (2.4% vs. 0.0%), and median commission rates decreased (61.0% vs. 55.0%). In terms of individual performance for accuracy, five-of-six participants increased accuracy from Trial 1 to Trial 2, when target exposure duration was longer. While all six participants decreased accuracy from Trial 2 to Trial 3, when target exposure duration was shortened, four-of-six participants had higher accuracy rates in Trial 3 compared to Trial 1 (including all three PD participants). In terms of individual performance for omission, two-of-six participants decreased omission rates from Trial 1 to Trial 2, with two remaining at 0.0% in both trials. One-of-six participants increased their omission rate from Trial 2 to Trial 3, with two decreasing their omission rates and three remaining at 0.0% in both trials. Comparing omission rates from Trial 1 to Trial 3, three-of-six participants had lower omission rates (including two-of-three PD participants), with three-of-six participants remaining at 0.0% in both trials (including one-of-three PD participants). In terms of individual performance for commission, five-of-six participants decreased commission rates from Trial 1 to Trial 2. Five-of-six participants increased their commission rate from Trial 2 to Trial 3, when target exposure duration was shortened. Comparing commission rates from Trial 1 to Trial 3, four-of six participants had lower commission rates (including all three PD participants), with one-of-six participants increasing commission from Trial 1 to Trial 3.

Table 2 reports the proportion of participants whose accuracy, omission, and commission rates changed across trials. In terms of accuracy, a significant proportion of participants increased their accuracy rate from Trial 1 to Trial 2, yet a significant proportion of participants decreased their accuracy rate from Trial 2 to Trial 3. In terms of commission, a significant proportion of participants decreased their commission rate from Trial 1 to Trial 2, yet a significant proportion of participants increased their commission rate from Trial 2 to Trial 3. No significant proportional changes were observed for the omission rates across the trials.

## 4. Discussion

Findings from this brief feasibility field trial suggest that a non-anticipatory, random-action target system (RATS) holds promise to improve shooting performance among shooters with firearm experience. This three-round trial had participants shoot at randomly appearing targets at random time intervals. In Trial 1, target exposure time was 0.5 s, which increased to 0.7 s in Trial 2, and decreased back to 0.5 s in Trial 3. This field trial confirms that shooting accuracy generally increases, while omission and commission generally decrease, when shooters are presented with targets exposed for longer durations. However, in this field trial, repeated shooting at targets with shorter exposure duration (i.e., Trial 1 vs. Trial 3) showed higher median accuracy rates, lower median omission rages, and lower median commission rates. Furthermore, individual performance revealed that all PD participants improved accuracy rates and reduced commission rates from Trial 1 to Trial 3, with two-of-three PD participants reducing omission rates from Trial 1 to Trial 3. Taken together, these findings support that PD shooters who need to make split-second decisions before discharging their firearm, while maintaining accuracy, improved their performance in a brief three-round field trial (i.e., countering the SAT). This is an encouraging finding in that PD participants, when presented with random targets in Trial 3, were both more accurate and able to discern when to fire at a target (reduction in errors of commission) compared to Trial 1. These data suggest the feasibility of the RATS to provide non-anticipatory training to PD and other military or tactical shooters, which may help them to better discern when to fire, and do so accurately, when forced to make split-second decisions in the field or combat situations. However, additional trials are needed with the RATS to replicate these findings among a larger and more diverse set of participants, who train with the RATS consistently, over longer durations.

The increased shooting performance from Trial 1 to Trial 2 supports that the presentation of randomly appearing targets with longer display times may benefit individuals who have high SAT in cognitively demanding environments [15]. It is hypothesized that by training with slightly longer target exposure times, then progressively decreasing target exposure times, shooters may be able to better balance reaction speed and accuracy, which are factors that impact decision making based on visual search [40]. Although there were no physiological data collected from participants to confirm this trade-off, it can be hypothesized that the change in target exposure duration allowed participants to improve their ability to switch between visual divergence and convergence when performing the visual search of the target [41]. In future trials, additional psychological and cognitive performance data should be collected to identify the profiles of shooters who perform better in terms of accuracy, omission, and/or commission over time.

One of the primary strengths of this feasibility field trial was that shooters were allowed to use a firearm with which they were most comfortable, which has been shown to increase shooting accuracy [42]. Another strength was the novel use of randomized target presentation, including randomization by time, position, and number of targets exposed. Relative to other static or anticipatory targets, this non-anticipatory RATS is more likely to mimic a “real-world” situation where the shooter might not know the number of potential targets or exactly where or when targets might appear [22,43]. All participants completed this single-day trial on the same day; therefore, external factors such as environmental conditions were not seen as a confounding factor across participants. Additionally, because the trial was brief and only consisted of three rounds (i.e., each participant fired a total of three magazines total in one day), participant fatigue was minimized and should not have impacted shooting performance. However, there were limitations of the study, which must be acknowledged and can guide future trials.

The small sample size, with limited types of shooters, was a limitation of the study. This small sample may have limited the ability to detect significant changes within and across participants in this three-round field trial. While including retired police officers and competitive shooters was appropriate for a feasibility trial, future studies would benefit from diversifying participants in terms of gender (e.g., including female participants), shooter type (e.g., active police officers, active military, lay public shooters), and shooting proficiency (e.g., including more shooters with novice or intermediate proficiency). Additional information about trial participants’ previous training and shooting habits should be collected, and accounted for in analyses, for additional context. Another potential shortcoming of the study was the lack of a baseline shooting accuracy rate using a static target. Establishing a baseline in future shooting trials may be beneficial to better understand the shooting accuracy of participants, which could be compared to shooting performance using the RATS. Similarly, this was a single-group trial, with no comparison group, which limits the ability to verify that shooting performance improvements were attributed to the RATS and not other confounders. However, the shooting performance improvements observed in this field trial from Trial 1 to Trial 3 may be attributed to learning effects [44], which is suggestive of potential longer-term benefits with increased and ongoing exposure to the RATS. This field trial was performed during a short period on a single day, which was insufficient to definitively identify the immediate and sustained training benefits of using the RATS for shooting performance. Future trails should be longitudinal in nature, which would allow participants to train with the RATS over time and more objectively assess the training potential for shooting accuracy, omission, and commission. Such trials would allow for the identification of training frequency and duration on shooting performance. Utilizing the G*Power parameters and data from the current study (see Methods section), it is estimated that 82 participants would be needed to detect significant improvements in a two-group randomized trial (i.e., 41 in each intervention arm). While the RATS can have tactical training advantages over other static or traditional target systems, costs and maintenance requirements associated with such dynamic systems should be considered.

## 5. Conclusions

Findings from this brief feasibility field trial were encouraging because participants generally increased accuracy while decreasing errors of omission and commission across the three rounds. Improvements in shooting performance were especially noted among PD participants, which highlights the potential utility of the RATS for tactical training to offset SAT. The adoption of the RATS into tactical training protocols may have promising practical application for law enforcement and military personnel by challenging shooters to react swiftly and accurately to unpredictable and non-anticipatory stimuli. By empowering shooters to decrease reaction times and increase shooting accuracy, this innovative system has the potential to advance training methodologies and enhance operational effectiveness in high-pressure environments and settings. To better understand the potential benefits of the RATS and its applications for tactical and real-world scenarios, additional more robust trials are needed. Future trials should engage larger samples of diverse shooters and incorporate longitudinal data collection with comparison groups to assess the short-term and sustained effectiveness of using the RATS for increasing shooting performance.

## Figures and Tables

**Table 1:** Participant characteristics and shooting performance across trials.

ID.	Age	Experience Level	Accuracy			Omission			Commission		
			Trial 1	Trial 2	Trial 3	Trial 1	Trial 2	Trial 3	Trial 1	Trial 2	Trial 3
PD1	58	A	23.1%	76.9%	30.8%	0.0%	7.1%	0.0%	76.9%	28.6%	69.2%
PD2	57	A	42.1%	100.0%	45.0%	5.0%	50.0%	0.0%	60.0%	50.0%	55.0%
PD3	50	A	40.0%	45.0%	45.0%	4.8%	0.0%	0.0%	61.9%	55.0%	55.0%
CS1	45	A	60.0%	60.0%	50.0%	0.0%	0.0%	0.0%	40.0%	40.0%	50.0%
CS2	47	A	75.0%	100.0%	60.0%	14.3%	0.0%	9.1%	35.7%	0.0%	45.5%
CS3	58	I	33.3%	80.0%	40.0%	0.0%	0.0%	0.0%	66.7%	20.0%	60.0%
Median			41.1%	78.5%	45.0%	2.4%	0.0%	0.0%	61.0%	34.3%	55.0%
Interquartile Rank [25]			[30.8%]	[56.3%]	[38.0%]	[0.0%]	[0.0%]	[0.0%]	[38.9%]	[15.0%]	[48.9%]
Interquartile Rank [75]			[63.8%]	[100.0%]	[52.5%]	[7.3%]	[17.9%]	[2.3%]	[69.2%]	[51.3%]	[62.3%]

PD = Police Department; CS = Competitive Shooter; A = Advanced; I = Intermediate.

**Table 2:** Sign-rank tests to identify changes in accuracy, omission, and commission across trials (n = 6).

Trial 1 vs. Trial 2				
	**Decrease**	**Increase**	**Same**	
Accuracy	0 (0.0%)	5 (83.3%)	1 (16.7%)	z = −2.02, *p* < 0.05
Omission	2 (33.3%)	2 (33.3%)	2 (33.3%)	z = −0.37, NS
Commission	5 (83.3%)	0 (0.0%)	1 (16.7%)	z = −2.02, *p* < 0.05
**Trial 2 vs. Trial 3**				
	**Decrease**	**Increase**	**Same**	
Accuracy	5 (83.3%)	0 (0.0%)	1 (16.7%)	z = −2.03, *p* < 0.05
Omission	2 (33.3%)	1 (16.7%)	3 (50.0%)	z = −0.54, NS
Commission	0 (0.0%)	5 (83.3%)	1 (16.7%)	z = −2.02, *p* < 0.05
**Trial 1 vs. Trial 3**				
	**Decrease**	**Increase**	**Same**	
Accuracy	2 (33.3%)	4 (66.7%)	0 (0.0%)	z = −0.11, NS
Omission	3 (50.0%)	0 (0.0%)	3 (50.0%)	z = −1.60, NS
Commission	4 (66.7%)	2 (33.3%)	0 (0.0%)	z = −0.11, NS
NS = Not Significant				

## Data Availability

The data presented in this study are available on request from the corresponding author.
